# Development and internal validation of machine learning models for identifying suicidal behavior in inpatients with mood disorders

**DOI:** 10.3389/fpsyt.2026.1950186

**Published:** 2026-10-05

**Authors:** Sixiang Liang, Qitong Jiang, Jinhe Zhang, Yannan Deng, Yuxuan Xu, Guangwei Zhao, Xinyu Liu, Yujie Xing, Hongye Yu, Chen Hu, Sha Sha

**Affiliations:** 1 The National Clinical Research Center for Mental Disorders & Beijing Key Laboratory of Mental Disorders, Beijing Anding Hospital, Capital Medical University & the Advanced Innovation Center for Human Brain Protection, Capital Medical University, Beijing, China; 2 Department of Clinical Psychology, Shandong Provincial Hospital Affiliated to Shandong First Medical University, Shandong, China

**Keywords:** calibration, internal validation, machine learning, mood disorders, SHAP, suicidal behavior

## Abstract

**Background:**

Suicidal behavior (SB) is a major concern in mood disorders. Models developed from retrospective records are vulnerable to ambiguous targets, data leakage, and inappropriate handling of class imbalance. We developed and internally validated a model to identify the risk of suicidal behavior in patients with mood disorders upon admission.

**Methods:**

The dataset included 1, 099 inpatients with ICD-10 mood disorders admitted to Beijing Anding Hospital in 2022. Exclusion of 20 records with more than 20% row-level missingness and one record without an outcome left 1, 078 patients, including 126 with SB. Current SB comprised documented suicidal ideation, planning, or attempt within the three months before index admission, including the admission assessment. Previous SB and previous suicide attempt (SA) referred to events more than three months before admission. Elastic Net selected 14 predictors from 65 candidates in the development set. We compared 13 classifiers from six model families. All preprocessing and resampling were confined to training folds, and evaluation used only observed patients. Assessment included repeated fivefold cross-validation, probability calibration, bootstrap confidence intervals, precision-recall and decision-curve analyses, clinical baselines, and sensitivity analyses.

**Results:**

The analytic cohort comprised 952 SB-negative and 126 SB-positive patients (11.69%). The development set contained 864 patients (97 SB-positive), and the observed test set contained 214 patients (29 SB-positive). Calibrated linear discriminant analysis (LDA) provided the most consistent balance of discrimination and calibration. In repeated cross-validation, mean AUC was 0.721 ± 0.052, mean AUPRC was 0.268 ± 0.060, and mean Brier score was 0.095. In the observed test set, AUC was 0.704 (0.597–0.803), AUPRC was 0.283 (0.198–0.453), and Brier score was 0.111. At the training-derived threshold of 0.111, sensitivity was 0.552 and specificity was 0.659. The leading SHAP contributions were onset-polarity category, previous SA, age, and previous SB, but all attributions were associational.

**Conclusions:**

Model performance was acceptable. These internally validated findings are exploratory and require prospective assessment, standardized outcome measurement, and external validation before clinical use.

## 1 Introduction

Suicide is a major global public-health problem. The World Health Organization estimates that approximately 727, 000 people die by suicide each year, and many more attempt suicide (1). Patients with mood disorders experience a substantial burden of suicidal ideation, planning, and attempts, although estimates vary across diagnostic and clinical settings (2, 3).

Suicidal behavior in mood disorders reflects demographic, clinical, psychosocial, biological, and health-service factors (4–8). Previous suicide attempts and other historical indicators have a suggestive effect on the current suicide risk of patients (9–14). Identifying patients with a high burden of recent suicidal behavior may support timely, structured assessment and closer monitoring during hospitalization.

Previous studies have used registry, demographic, psychometric, genetic, and laboratory data to identify suicide risk (15–19). Machine-learning models can capture nonlinear associations and interactions, but they can also overfit, produce poorly calibrated probabilities, and be difficult to interpret when outcome events are uncommon (15, 20, 21). ROC AUC alone does not show how well a model identifies the minority class, estimates absolute risk, or supports clinical decisions. Evaluation should therefore include precision-recall performance, calibration, uncertainty, and decision-curve analysis, together with comparison against simple clinical baselines (22–26).

Shapley additive explanations (SHAP) can describe how predictors contribute to individual model outputs (27). Related work in major depressive disorder has combined conventional statistical characterization with interpretable machine learning (28). However, SHAP values describe model associations rather than causal mechanisms, and importance rankings can be unstable in modest samples. Transparent evaluation therefore requires leakage-free preprocessing, explicit handling of class imbalance, calibration, and repeated assessment of explanation stability.

We used a single-center inpatient cohort to examine whether demographic characteristics, clinical history, and routine endocrine and biochemical measurements available around admission could identify patients with suicidal behavior documented during the preceding three months. The clinical aim was to support prioritization for detailed risk assessment at admission. We compared 13 model families and several imbalance strategies, assessed incremental value beyond remote suicide history, and evaluated discrimination, minority-class performance, calibration, uncertainty, net benefit, and explanation stability.

## 2 Materials and methods

### 2.1 Study design and participants

This retrospective single-center study used de-identified inpatient medical-record data from Beijing Anding Hospital, Capital Medical University, for calendar year 2022. Eligible patients received an ICD-10 diagnosis within F30–F39, independently assigned by at least two attending psychiatrists. Exclusion criteria were comorbid schizoaffective disorder, personality disorder, intellectual disability, severe physical illness, or substance abuse. Records with more than 20% missing fields were excluded. The study was approved by the institutional ethics committee (R202409190355) and registered retrospectively (ChiCTR2400090041) in 2024, after data collection. Written authorization for anonymized research use of medical-record information was recorded.

The dataset initially contained 1, 099 records. The row-level missingness rule excluded 20 records, and one further record lacked an ascertainable current-SB outcome. Outcomes were not imputed. The analytic cohort therefore included 1, 078 patients, of whom 864 formed the development set and 214 formed the evaluation set. Event proportions were 11.23% and 13.55%, respectively. The partition was treated as random rather than exactly outcome-stratified.

### 2.2 Outcome and temporal definition

Current SB was defined as suicidal ideation, a suicide plan, or a suicide attempt documented within the previous three months, including at admission. Patients with any of these phenomena were classified as SB-positive; all others with an observed outcome were classified as SB-negative. Previous SB and previous SA were non-overlapping historical predictors and referred to events more than three months before admission. Routine laboratory results were available within 72 hours after admission. The model therefore identifies recent SB at admission and does not estimate prospective risk of a later attempt or suicide death.

### 2.3 Candidate predictors and missing data

After removal of the identifier and outcome, current suicide attempt and current non-suicidal self-injury were excluded because they referred to the same three-month window as the outcome and could introduce target leakage. Sixty-five candidate predictors remained, covering demographics, prior self-harm, coded diagnosis and onset polarity, illness and hospitalization characteristics, medical comorbidity, and routine laboratory measurements. The de-identified dataset did not retain a reliable mapping for major depressive disorder, bipolar I disorder, and bipolar II disorder. Diagnosis was therefore excluded from the final 14-predictor set, and subtype analyses were not performed.

Onset polarity was defined as the polarity of the first documented affective episode and was coded as depressive onset (1), manic onset (2), or not recorded/not applicable. Per-variable missingness was calculated before imputation and is reported in **Supplementary Table 1**. Most candidate variables had 0–10% missingness; ACTH had 18.0% missingness. The onset-polarity field had 33.3% unrecorded values and was treated as an explicit ‘not recorded/not applicable’ category rather than numerically median-imputed, because non-recording may be structurally related to diagnosis. Continuous predictors were median-imputed and standardized; categorical predictors were assigned an explicit missing category and one-hot encoded. Every imputer, encoder, and scaler was fitted only on the relevant training fold and then applied to the held-out fold or test set. The outcome was never imputed. A sensitivity analysis excluded both onset polarity and ACTH.

### 2.4 Statistical characterization and feature selection

Continuous variables were summarized as median (interquartile range) and compared using the Mann–Whitney U test; rank-biserial correlation and the common-language effect size were reported. Categorical variables were summarized as n (%) and compared using χ² tests with Cramér’s V. Benjamini–Hochberg correction controlled the false-discovery rate across all 65 candidate comparisons.

Feature selection was conducted only in the 864-patient development set and was completed before test-set evaluation. To limit overfitting given the 97 SB events in the development data, Elastic Net reduced the 65 candidates to 14 predictors: ACTH, ALT, AST, complement C4, CK-MB, age, cortisol, creatinine, high-density lipoprotein, onset-polarity category, previous SA, previous SB, progesterone, and testosterone. The Elastic Net used an L1/L2 mixing ratio of 0.25 and an inverse regularization strength of 0.0178. We compared this selection with rankings from mutual information, recursive feature elimination, and cross-fitted permutation importance. Bootstrap resampling assessed rank stability (**Supplementary Table 2**) (29). The methods did not select exactly the same variables, so the retained set should be regarded as uncertain rather than definitive.

### 2.5 Model development, imbalance strategies, and internal validation

Thirteen classifiers were compared: Elastic-Net logistic regression, linear discriminant analysis (LDA), Gaussian naïve Bayes, k-nearest neighbors, radial-basis-function support vector machine, multilayer perceptron, decision tree, random forest, AdaBoost, histogram gradient boosting, XGBoost, LightGBM, and CatBoost. Model fitting used fixed settings, with hyperparameters and software versions reported in **Supplementary Table 3**.

The cohort included 126 SB-positive and 952 SB-negative patients, representing moderate class imbalance. We compared observed-prior modeling, equal-prior or cost-sensitive LDA, class-weighted Elastic-Net logistic regression, and SMOTE-NC. SMOTE-NC was included as a sensitivity analysis because the predictors included both categorical and continuous variables. Resampling, when used, occurred only within training folds; the test set remained unchanged. Strategy selection considered AUC, AUPRC, and Brier score because oversampling and cost weighting can alter calibration.

Stability was assessed by fivefold stratified cross-validation repeated five times in the development set. Within each outer training fold, preprocessing was re-fitted and threefold internal calibration was used where applicable. The final selected model was refitted on the complete development set and sigmoid-calibrated using fivefold internal predictions. The operating threshold (0.111) maximized Youden’s J on out-of-fold development predictions and was locked before test evaluation. The 214 observed test patients were evaluated once without resampling.

### 2.6 Performance, clinical baselines, calibration, and explanation

We report ROC AUC, AUPRC, Brier score, accuracy, sensitivity, specificity, precision, F1 score, and MCC. Stratified nonparametric bootstrap resampling (2, 000 replicates) provided 95% confidence intervals on the observed test set. Calibration was summarized by a calibration plot, intercept, and slope. Decision-curve analysis used calibrated probabilities at thresholds from 0.02 to 0.50. Paired bootstrap differences compared the selected model with leading alternatives. Accuracy was not used as the model-selection criterion.

Clinical incremental value was examined against (1) a logistic model containing previous SA alone and (2) a parsimonious model containing previous SA, previous SB, age, and onset-polarity category. Sensitivity analyses removed previous SA and previous SB, removed onset polarity, and removed predictors with more than 15% missingness (onset polarity and ACTH).

For the final LDA model, SHAP values were computed on the observed test patients on the log-odds scale. One-hot levels were aggregated to their source predictor. Orange indicates higher feature values and blue indicates lower values; an explicit missing category is coded lower than recorded polarity categories only for visualization. SHAP ranking stability was assessed across 200 bootstrap refits. SHAP values are interpreted as model associations and not causal effects.

### 2.7 Sample-size adequacy and software

A *post hoc* sample-size calculation used 14 predictor parameters, a prevalence of 0.117, an anticipated C-statistic of 0.721, and a target shrinkage of 0.90. The calculation estimated a minimum of 1, 850 patients and 217 events. The available 1, 078 patients and 126 events fell below this estimate. We therefore used penalization, limited the number of predictors, repeated resampling, and reported interval estimates.

Analyses used Python 3.12 with pandas 3.0, scipy 1.18, scikit-learn 1.9, imbalanced-learn 0.14, XGBoost 3.4, LightGBM 4.7, CatBoost 1.2, statsmodels 0.14, and SHAP-compatible linear attribution routines. Statistical tests were two-sided. Reproducible analysis scripts and the fitted-model specification are available from the corresponding author subject to institutional data-governance requirements. Reporting was aligned with TRIPOD+AI (26).

## 3 Results

### 3.1 Cohort and class accounting

Among 1, 078 analyzed patients, 126 (11.69%) had current SB within the three-month window and 952 (88.31%) did not. The development set contained 97 events among 864 patients; the observed test set contained 29 events among 214 patients. No evaluation patient was duplicated or synthesized. **Figure 1** shows the exclusions, class counts, and bootstrap stability of the final SHAP ranking. **Table 1** describes the 14 retained predictors. Younger age, previous SA, previous SB, onset-polarity category, AST, CK-MB, creatinine, and testosterone remained different after false-discovery-rate correction. Marginal effect sizes were generally small to modest.

**Figure 1:**
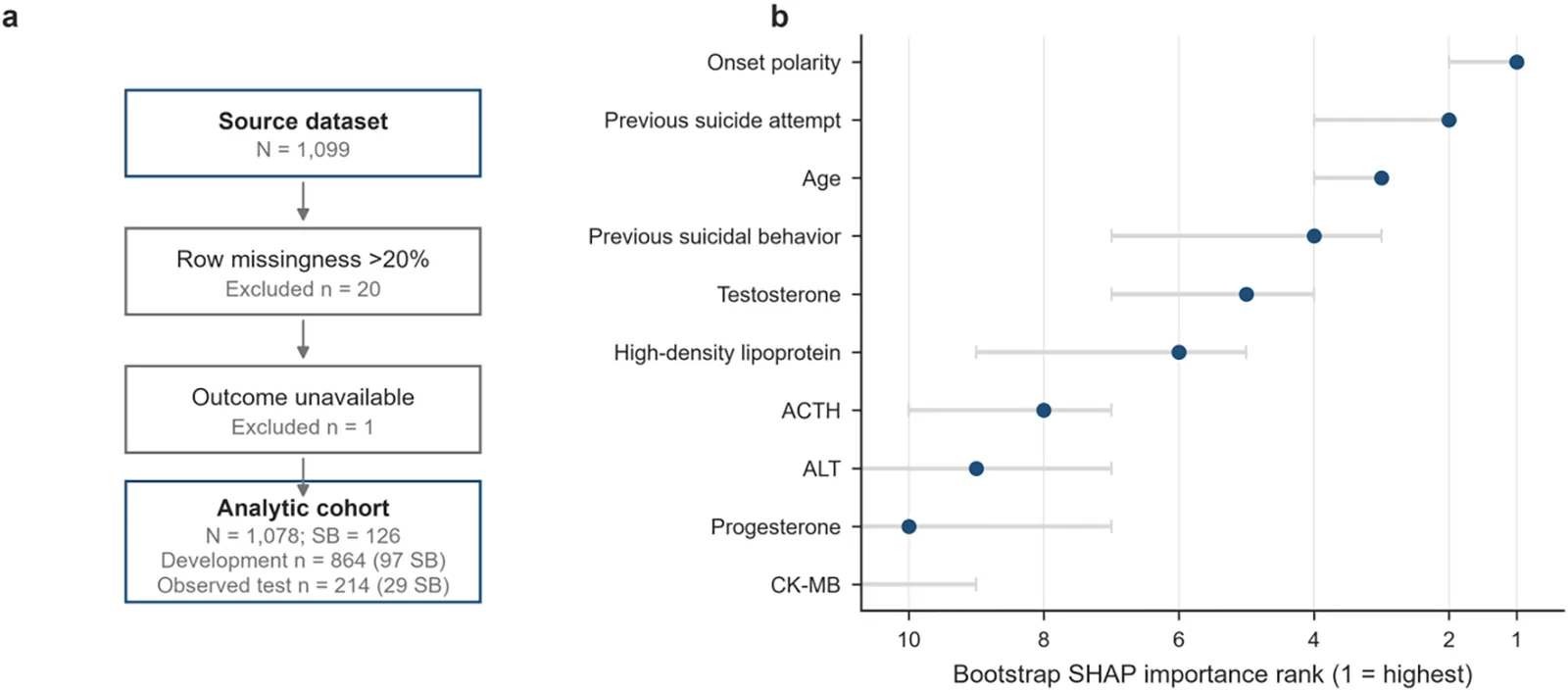
Cohort flow and explanation stability. **(a)** Accounting of observed patients and classes after applying the prespecified missingness and outcome rules. **(b)** Median and interquartile range of SHAP importance rank across 200 bootstrap refits; rank 1 is most important.

**Table 1:** Distribution and effect size of retained predictors in the analytic cohort.

Predictor	Overall (N = 1, 078)	SB-N (n=952)	SB (n=126)	Effect size	FDR-adjusted p
ACTH	32.20 (21.20–48.05)	32.70 (22.05–48.40)	31.40 (17.50–43.50)	r_rb -0.147; CL 0.426	0.0568
ALT	15.90 (10.10–26.00)	16.20 (10.25–26.10)	13.10 (9.50–23.85)	r_rb -0.117; CL 0.442	0.12
AST	18.90 (15.60–25.70)	19.00 (15.70–26.10)	17.75 (14.40–22.53)	r_rb -0.154; CL 0.423	0.0233
Complement C4	0.20 (0.16–0.25)	0.20 (0.16–0.25)	0.20 (0.16–0.24)	r_rb -0.061; CL 0.469	0.519
CK-MB	0.00 (0.00–13.00)	0.00 (0.00–13.00)	0.00 (0.00–11.00)	r_rb -0.161; CL 0.420	0.0107
Age	31.00 (21.00–46.00)	32.00 (21.00–47.00)	24.50 (19.00–39.00)	r_rb -0.202; CL 0.399	0.00239
Cortisol	17.26 (12.53–22.10)	17.36 (12.78–22.19)	16.04 (10.80–21.17)	r_rb -0.116; CL 0.442	0.12
Creatinine	69.00 (59.00–80.00)	69.00 (60.00–80.50)	64.00 (58.00–73.75)	r_rb -0.167; CL 0.417	0.0135
High-density lipoprotein	1.20 (1.00–1.40)	1.20 (1.00–1.40)	1.20 (1.00–1.40)	r_rb 0.082; CL 0.541	0.378
Onset-polarity category	mania 482 (44.7%); not recorded/not applicable 359 (33.3%); depression 237 (22.0%)	mania 397 (41.7%); not recorded/not applicable 326 (34.2%); depression 229 (24.1%)	mania 85 (67.5%); not recorded/not applicable 33 (26.2%); depression 8 (6.3%)	Cramér’s V 0.179	6.83e-07
Previous SA	497 (46.1%) positive	409 (43.0%) positive	88 (69.8%) positive	Cramér’s V 0.170	6.83e-07
Previous SB	199 (18.5%) positive	152 (16.0%) positive	47 (37.3%) positive	Cramér’s V 0.173	6.83e-07
Progesterone	0.56 (0.36–0.83)	0.56 (0.35–0.83)	0.57 (0.37–0.87)	r_rb 0.053; CL 0.527	0.568
Testosterone	78.33 (26.43–354.96)	106.81 (26.56–363.40)	34.09 (25.07–279.87)	r_rb -0.162; CL 0.419	0.0164

Values are median (IQR) unless otherwise indicated. CL, common-language effect size (probability that a randomly selected SB patient has a higher value than a randomly selected SB-N patient); r_rb, rank-biserial correlation. Laboratory units are those recorded by the hospital laboratory information system.

### 3.2 Model comparison and imbalance handling

Gaussian naïve Bayes had the highest mean uncalibrated AUC in repeated development-set cross-validation. Its test-set discrimination and calibration, however, were less consistent. LDA showed the best overall balance of AUC, AUPRC, Brier score, and test-set performance and was selected for calibration and interpretation. Paired bootstrap comparisons between calibrated LDA and Gaussian naïve Bayes, CatBoost, or random forest all produced AUC-difference intervals that included zero. The detail mentioned above shown in **Table 2** and **Figures 2**, **3**.

**Table 2:** Repeated cross-validation and observed-test performance of 13 model families.

Model	CV AUC	CV AUPRC	Test AUC	Test AUPRC	Test Brier
Gaussian naive Bayes	0.725 ± 0.065	0.273 ± 0.059	0.678	0.268	0.166
Linear discriminant analysis	0.702 ± 0.071	0.270 ± 0.056	0.681	0.284	0.110
Random forest	0.701 ± 0.060	0.239 ± 0.050	0.649	0.280	0.114
Elastic-net logistic regression	0.697 ± 0.062	0.243 ± 0.047	0.669	0.259	0.112
CatBoost	0.696 ± 0.064	0.229 ± 0.054	0.614	0.306	0.115
AdaBoost	0.693 ± 0.067	0.206 ± 0.048	0.634	0.196	0.117
XGBoost	0.682 ± 0.055	0.209 ± 0.038	0.609	0.240	0.119
Histogram gradient boosting	0.667 ± 0.059	0.198 ± 0.043	0.587	0.223	0.124
K-nearest neighbours	0.662 ± 0.057	0.216 ± 0.040	0.632	0.285	0.114
LightGBM	0.660 ± 0.057	0.191 ± 0.035	0.577	0.268	0.122
Decision tree	0.648 ± 0.078	0.198 ± 0.047	0.597	0.172	0.126
RBF support vector machine	0.589 ± 0.063	0.199 ± 0.045	0.508	0.182	0.116
Multilayer perceptron	0.484 ± 0.043	0.112 ± 0.013	0.658	0.229	0.135

Cross-validation values are mean ± SD across fivefold cross-validation repeated five times. Values are for fixed reproducible model settings before final sigmoid calibration.

**Figure 2:**
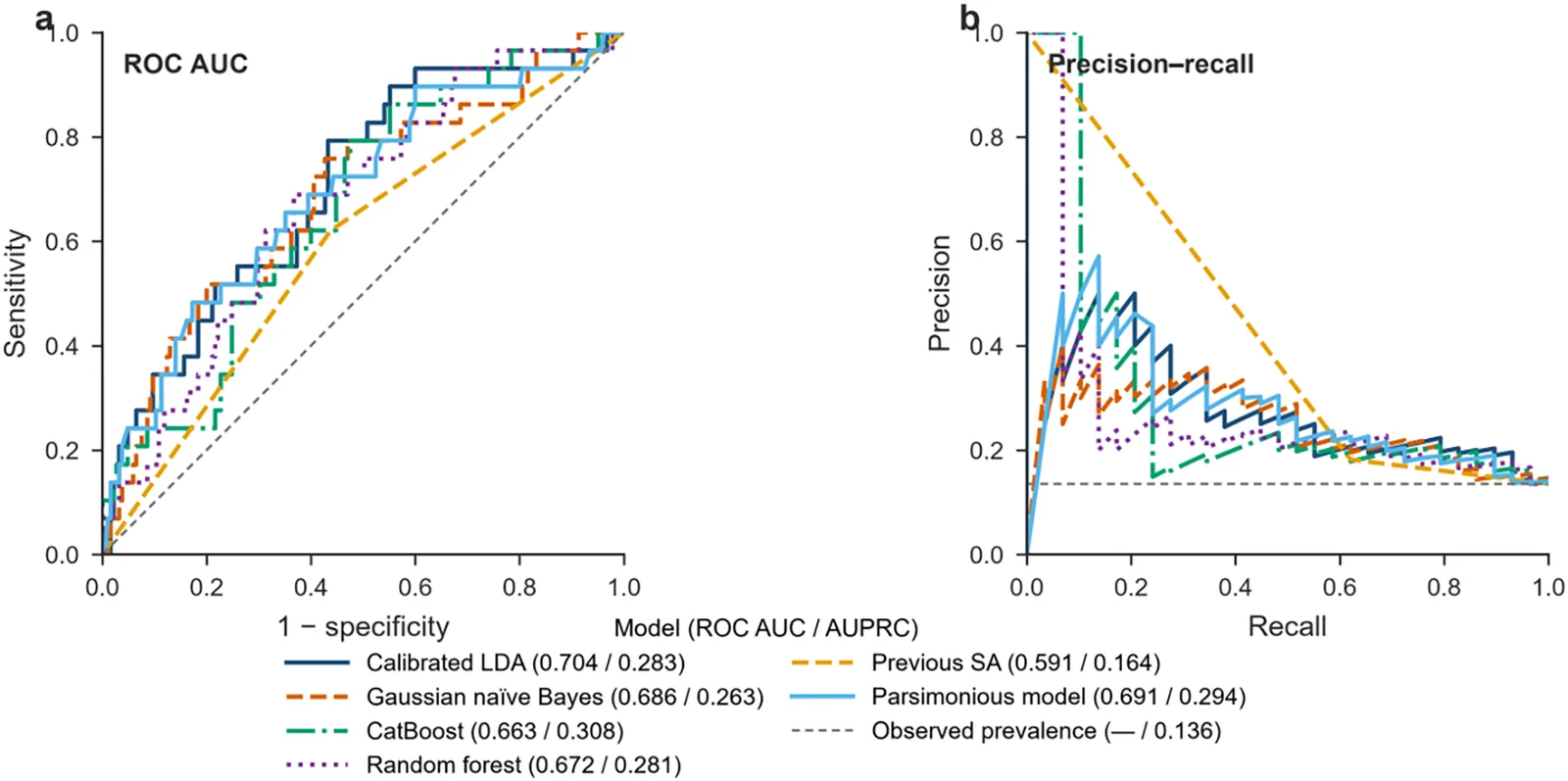
Discrimination and minority-class performance in the observed test set. **(a)** Receiver-operating-characteristic curves. **(b)** Precision–recall curves; the horizontal dashed line is the observed event prevalence (13.55%). Legend values are ROC AUC/AUPRC. No synthetic observations were evaluated.

**Figure 3:**
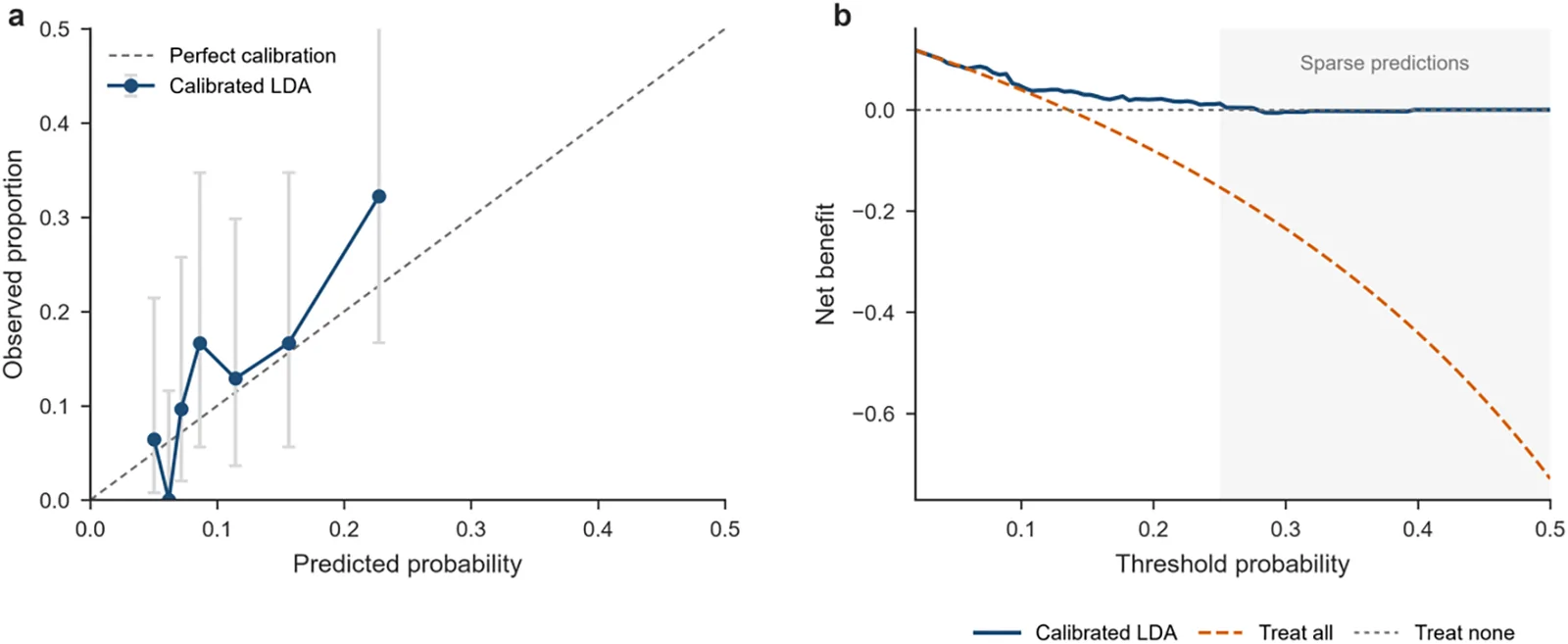
Calibration and decision-curve analysis of calibrated LDA on observed test patients. **(a)** Observed event proportions by quantile of predicted risk with exact binomial 95% confidence intervals; the dashed diagonal indicates perfect calibration. **(b)** Net benefit relative to treat-all and treat-none strategies.

Models fitted to the observed class distribution achieved cross-validated AUC 0.719, AUPRC 0.281, and Brier score 0.097. Equal-prior LDA, class-weighted logistic regression, and fold-restricted SMOTE-NC yielded Brier scores of 0.215, 0.208, and 0.210, respectively, without consistent gains in discrimination. The final probability model therefore used neither oversampling nor class weighting. Its operating threshold was selected from out-of-fold development predictions after model fitting. **Table 3**, Panel C reports the full comparison.

**Table 3:** Final-model performance, clinical baselines, sensitivity analyses, and class-imbalance strategies.

Analysis/model	ROC AUC	AUPRC	Brier score	Additional results/interpretation
Panel A. Final calibrated LDA				
Repeated fivefold CV ×5	0.721 ± 0.052	0.268 ± 0.060	0.095 ± 0.004	Mean ± SD
Observed test set	0.704 (0.597–0.803)	0.283 (0.198–0.453)	0.111 (0.105–0.118)	N=214; 29 events
Locked operating point	—	—	—	Threshold 0.111; sensitivity 0.552; specificity 0.659; precision 0.203; F1 0.296; MCC 0.150
Calibration	—	—	—	Intercept 0.572 (-0.807–1.951); slope 1.164 (0.488–1.840)
Panel B. Clinical baselines and sensitivity analyses				
Previous SA only	0.591	0.164	0.116	Clinical baseline
Four-variable parsimonious model	0.691	0.294	0.111	Previous SA, previous SB, age, onset polarity
Without previous SA and SB	0.661	0.209	0.116	History-exclusion sensitivity
Without onset polarity	0.662	0.262	0.112	Missingness sensitivity
Without onset polarity and ACTH	0.647	0.254	0.113	Predictors with >15% missingness removed
Panel C. Class-imbalance strategies				
No correction (LDA, observed priors)	0.719/0.705	0.281/0.285	0.097/0.111	CV/observed test
Cost-sensitive/equal-prior LDA	0.724/0.691	0.272/0.283	0.215/0.213	CV/observed test
Class-weighted logistic regression	0.717/0.682	0.261/0.263	0.208/0.207	CV/observed test
SMOTE-NC within training only	0.703/0.648	0.230/0.215	0.210/0.213	CV/observed test

Values in parentheses in Panel A are 95% stratified bootstrap confidence intervals based on 2, 000 resamples. For Panel C, each cell reports repeated-cross-validation mean/observed-test value. SA, suicide attempt; SB, suicidal behavior.

### 3.3 Final model performance, calibration, and incremental value

After sigmoid calibration, repeated cross-validation yielded mean AUC 0.721 ± 0.052, AUPRC 0.268 ± 0.060, and Brier score 0.095 ± 0.004. On the 214 observed test patients, AUC was 0.704 (0.597–0.803), AUPRC 0.283 (0.198–0.453), and Brier score 0.111. At the locked threshold 0.111, the confusion matrix was TN = 122, FP = 63, FN = 13, and TP = 16. Calibration intercept was 0.572 (95% CI -0.807 to 1.951) and slope 1.164 (95% CI 0.488 to 1.840); intervals were wide because only 29 test events were available.

The previous-SA-only baseline yielded AUC 0.591; the four-variable parsimonious logistic model yielded AUC 0.691. The final model therefore improved AUC by 0.112 over previous SA alone but by only 0.013 over the parsimonious model. After excluding previous SA and previous SB, AUC decreased to 0.661 and AUPRC to 0.209. Excluding onset polarity alone yielded AUC 0.662; excluding both onset polarity and ACTH yielded AUC 0.647. These analyses indicate incremental information beyond suicide history but also substantial reliance on historical and onset-polarity fields. The detail mentioned above shown in **Figures 2**, **3**.

### 3.4 Model explanation

Mean absolute SHAP values ranked onset-polarity category first, followed by previous SA, age, previous SB, testosterone, and high-density lipoprotein. Across 200 bootstrap refits, median ranks (IQR) were 1 (1–2) for onset polarity, 2 (2–4) for previous SA, 3 (3–4) for age, and 4 (3–7) for previous SB. Lower-ranked laboratory features showed wider rank variation. The beeswarm direction agreed with conventional group comparisons: recorded manic-onset category and positive previous-SA/SB values contributed toward higher modeled log odds, whereas older age and higher testosterone tended to contribute toward lower modeled log odds. These are conditional model associations and do not establish causation. The detail of SHAP shown in **Figure 4**.

**Figure 4:**
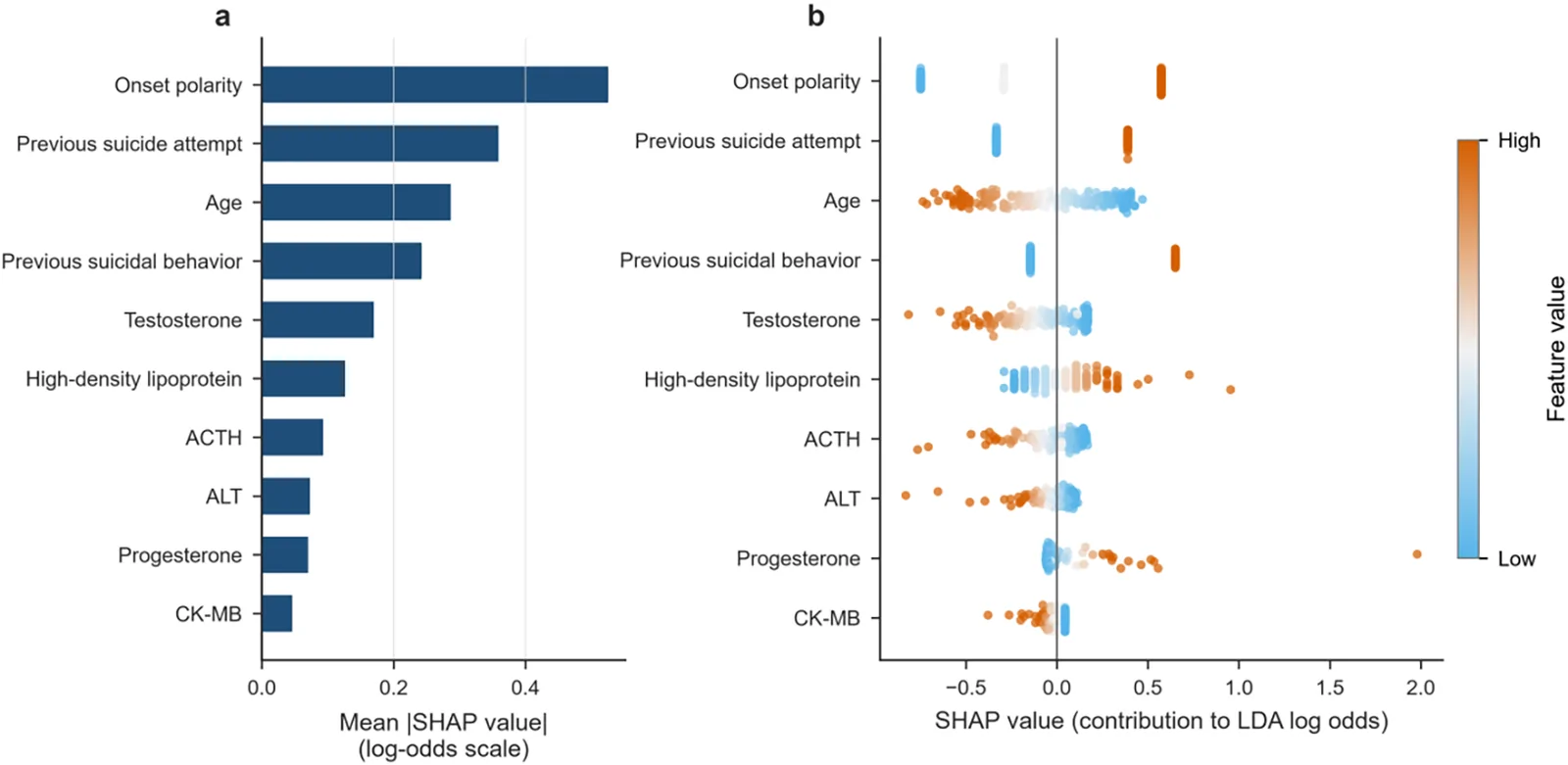
Global and directional SHAP explanation of the final LDA model in the 214 observed test patients. **(a)** Mean absolute SHAP values for the ten leading source predictors after aggregation of one-hot levels. **(b)** Distribution and direction of patient-level SHAP values; positive values increase modeled log odds of current SB within the three-month window, orange denotes higher feature values, blue denotes lower values, and missing values are shown at the midpoint of the color scale. For onset polarity, the underlying codes are −1=not recorded/not applicable, 1=depressive onset, and 2=manic onset.

## 4 Discussion

The calibrated LDA model showed moderate discrimination and the most consistent balance of repeated-validation stability, test-set performance, precision-recall performance, and calibration. Complex boosting models showed no statistically clear advantage.

Previous SA and previous SB, both occurring more than three months before admission, were among the strongest predictors. This is consistent with evidence that a history of suicidal behavior remains clinically informative (9–14). The previous-SA-only baseline had limited discrimination, whereas adding previous SB, age, and onset polarity brought performance close to that of the full model. A small set of historical and relatively stable characteristics available at admission may therefore help identify patients who need more intensive suicide-risk assessment and monitoring during hospitalization. The additional laboratory variables provided only modest incremental information.

Within this cohort, recorded manic onset contributed more strongly to a higher modeled probability of SB and was more common among SB-positive patients. This direction differs from several studies restricted to BD, which associated depressive onset with suicide attempts (30–35). One plausible explanation is that onset polarity partly acted as a proxy for diagnostic subtype. A documented manic first episode is consistent with bipolar-spectrum illness rather than unipolar major depressive disorder (MDD), whereas a depressive first episode can occur in either MDD or bipolar disorder (BD). In a comparative cohort of 3, 284 adults with major mood disorders, suicidal ideation, suicide attempts, and suicide were all more prevalent in BD than in MDD (36). BD diagnosis also remained independently associated with suicidal acts (36). Thus, greater representation of bipolar-spectrum illness in the manic-onset category could have contributed to the higher modeled probability observed here. This between-diagnosis interpretation complements, rather than replaces, evidence linking depressive onset to suicide attempts within BD. However, diagnostic subtype could not be verified in the de-identified dataset, and 33.3% of onset-polarity values were unrecorded. Referral and coding patterns at this tertiary center may also have contributed. The lower AUC after excluding onset polarity confirms model reliance on this field but does not establish a causal or broadly generalizable association.

Laboratory variables provided less stable information than historical and demographic predictors. Although testosterone, high-density lipoprotein, ACTH, ALT, progesterone, and CK-MB were retained during feature selection, most showed small marginal contributions and unstable bootstrap rankings. Their apparent associations should therefore be interpreted cautiously. Measurements obtained within 72 hours of admission may reflect transient physiological conditions rather than stable risk markers. They may also be influenced by acute illness severity, recent treatment, fasting status, sex, age, and comorbidities. This interpretation is further limited by the absence of medication data, including lithium, antidepressants, antipsychotics, and agents affecting endocrine or metabolic measures, which prevented adjustment for treatment-related confounding. Inflammatory markers emphasized in earlier studies were also only partially represented in this dataset (37–39). Accordingly, these laboratory findings are best viewed as exploratory signals that require confirmation in prospectively standardized cohorts with detailed medication and sampling information.

Because only about 12% of patients were SB-positive, we tested equal-prior learning, class weighting, and SMOTE-NC. None improved discrimination while preserving calibration, and SMOTE-NC lowered test AUC and increased the Brier score. The final model therefore retained the observed class distribution. Its operating threshold was selected from development-set predictions and was not adjusted on the test set. This threshold remains provisional because the clinical consequences of false-positive and false-negative classifications were not assessed.

### 4.1 Strengths and limitations

A principal strength of this study is the structured development and evaluation of the SB identification model. We compared 13 classifiers and several class-imbalance strategies, then assessed the selected model using repeated cross-validation and an untouched observed test set. Evaluation covered discrimination, minority-class performance, calibration, bootstrap uncertainty, decision-curve analysis, clinical baselines, sensitivity analyses, and SHAP-rank stability. Non-overlapping temporal definitions, exclusion of concurrent target-adjacent variables, and fold-specific preprocessing reduced the risk of information leakage.

Several design and measurement limitations constrain interpretation. The study was retrospective, single-center, and restricted to one year of inpatient data, with retrospective registration and no external validation. The chart-derived outcome was not measured with a validated suicide instrument, and inter-rater reliability was unavailable. Standardized symptom severity, hopelessness, impulsivity, detailed medication exposure, and raw PSP scores were also unavailable. Missingness was high for onset polarity (33.3%) and ACTH (18.0%); fold-wise imputation cannot remove bias when missingness is informative. The absence of reliable diagnostic subtype mapping also prevented subgroup analysis.

Model uncertainty was also considerable. The 126 events fell below the *post hoc* adequacy estimate of 217, and agreement between feature selectors was incomplete. Residual dependence between predictors may have affected both selection and attribution. The test set contained only 29 events, producing wide confidence intervals and unstable calibration estimates. SHAP values describe conditional model associations and do not imply that changing a laboratory marker would alter suicidal behavior.

## 5 Conclusion

In this retrospective inpatient cohort, a calibrated LDA model using 14 routinely available predictors showed moderate ability to identify suicidal behavior occurring within the three months preceding index admission, and complex boosting models did not show a clear advantage. More remote suicide history, onset-polarity category, and age carried much of the model signal, with limited but detectable incremental information from the wider predictor set. The model should be regarded as exploratory and internally validated only; prospective standardized outcome ascertainment and independent external validation are required before clinical use.

## Data Availability

The raw data supporting the conclusions of this article will be made available by the authors, without undue reservation.
