# Supplementary material for: Development and internal validation of machine learning models for identifying suicidal behavior in inpatients with mood disorders
Source: Front Psychiatry. 2026 Oct 5;17:1950186. doi: 10.3389/fpsyt.2026.1950186 (PMC13678466; doi:10.3389/fpsyt.2026.1950186)
Supplement: Supplementary file 1 [file Table1.docx]

Supplemental Table 1.Basic information of test group after SMOTE

| Characteristics | | Overall | SB-N | SB | p-value |
| --- | --- | --- | --- | --- | --- |
|  |  | N = 378 | N = 189 | N = 189 |  |
| Age, median (p25 - p75) |  | 27 (19 - 43) | 32 (21 - 51) | 23.00 (18 - 35) | <0.001 |
| Hospitalizationdays, median (p25 - p75) |  | 28 (16 - 44) | 32 (22 - 49) | 22 (11 - 38) | <0.001 |
| gender, n (p%) |  |  |  |  | <0.001 |
| male |  | 170 (44.97%) | 101 (53.44%) | 69 (36.51%) |  |
| previous_sa, n (p%) |  |  |  |  | 0.003 |
|  |  | 193 (51.06%) | 82 (43.39%) | 111 (58.73%) |  |
| previous_sb, n (p%) |  |  |  |  | <0.001 |
|  |  | 106 (28.04%) | 30 (15.87%) | 76 (40.21%) |  |
| previous_nssi, n (p%) |  |  |  |  | <0.001 |
|  |  | 82 (21.69%) | 12 (6.35%) | 70 (37.04%) |  |
| income, n (p%) |  |  |  |  | <0.001 |
| ＜1000 |  | 7 (1.85%) | 7 (3.70%) | 0 (0.00%) |  |
| 1000-3000 |  | 2 (0.53%) | 2 (1.06%) | 0 (0.00%) |  |
| 3000-5000 |  | 22 (5.82%) | 11 (5.82%) | 11 (5.82%) |  |
| ＞5000 |  | 60 (15.87%) | 18 (9.52%) | 42 (22.22%) |  |
| NA |  | 287 (75.93%) | 151 (79.89%) | 136 (71.96%) |  |
| diagnosis, n (p%) |  |  |  |  | <0.001 |
| Depression |  | 167 (44.18%) | 71 (37.57%) | 96 (50.79%) |  |
| Mania |  | 33 (8.73%) | 1 (0.53%) | 32 (16.93%) |  |
| Bipolar Disorder |  | 140 (37.04%) | 87 (46.03%) | 53 (28.04%) |  |
| Mood Disorder |  | 38(10.06%) | 30 (15.88%) | 8 (4.23%) |  |
| Onset polarity, n (p%) |  |  |  |  | 0.034 |
| Depression |  | 115 (30.42%) | 67 (35.45%) | 48 (25.40%) |  |
| Mania |  | 263 (69.58%) | 122 (64.55%) | 141 (74.60%) |  |

Supplemental Table 2.Basic information of train group after SMOTE

| Characteristics | | Overall | SB-N | SB | p-value |
| --- | --- | --- | --- | --- | --- |
|  |  | N = 1,562 | N = 781 | N = 781 |  |
| Age, median (p25 - p75) |  | 28 (20 - 42) | 32 (21 - 46) | 25 (19 - 37) | <0.001 |
| Hospitalizationdays, median (p25 - p75) |  | 33 (22 - 46) | 33 (23 - 46) | 32 (22 - 46) | 0.191 |
| gender, n (p%) |  |  |  |  | <0.001 |
| male |  | 723 (46.29%) | 411 (52.62%) | 312 (39.95%) |  |
| previous_sa, n (p%) |  |  |  |  | <0.001 |
|  |  | 885 (56.66%) | 334 (42.77%) | 551 (70.55%) |  |
| previous_sb, n (p%) |  |  |  |  | <0.001 |
|  |  | 398 (25.48%) | 125 (16.01%) | 273 (34.96%) |  |
| previous_nssi, n (p%) |  |  |  |  | <0.001 |
|  |  | 265 (16.97%) | 103 (13.19%) | 162 (20.74%) |  |
| income, n (p%) |  |  |  |  | <0.001 |
| ＜1000 |  | 43 (2.75%) | 36 (4.61%) | 7 (0.90%) |  |
| 1000-3000 |  | 12 (0.77%) | 4 (0.51%) | 8 (1.02%) |  |
| 3000-5000 |  | 68 (4.35%) | 32 (4.10%) | 36 (4.61%) |  |
| ＞5000 |  | 208 (13.32%) | 87 (11.14%) | 121 (15.49%) |  |
| NA |  | 1,231 (78.81%) | 622 (79.64%) | 609 (77.98%) |  |
| diagnosis, n (p%) |  |  |  |  | <0.001 |
| Depression |  | 729 (46.67%) | 284 (36.36%) | 445 (56.98%) |  |
| Mania |  | 169 (10.82%) | 7 (0.90%) | 162 (20.74%) |  |
| Bipolar Disorder |  | 517 (33.10%) | 354 (45.33%) | 163 (20.87%) |  |
| Mood Disorder |  | 147 (9.42%) | 136 (17.41%) | 11 (1.41%) |  |

Supplemental Table 3. Basic information of Test group

| Characteristics | Overall | SB-N | SB | p-value |
| --- | --- | --- | --- | --- |
|  | N = 219 | N = 189 | N = 30 |  |
| age, median (p25 - p75) | 32 (21- 50) | 32 (21 - 51) | 23 (17 - 39) | 0.026 |
| gender, n (p%) |  |  |  | 0.041 |
| Male | 111 (50.68%) | 101 (53.44%) | 10 (33.33%) |  |
| previous_sa, n (p%) |  |  |  | 0.090 |
|  | 100 (45.66%) | 82 (43.39%) | 18 (60.00%) |  |
| previous_sb, n (p%) |  |  |  | 0.002 |
|  | 42 (19.18%) | 30 (15.87%) | 12 (40.00%) |  |
| previous_nssi, n (p%) |  |  |  | <0.001 |
|  | 21 (9.59%) | 12 (6.35%) | 9 (30.00%) |  |
| marriage, n (p%) |  |  |  | 0.913 |
| Single | 65 (29.68%) | 57 (30.16%) | 8 (26.67%) |  |
| Married | 72 (32.88%) | 60 (31.75%) | 12 (40.00%) |  |
| Divorced | 1 (0.46%) | 1 (0.53%) | 0 (0.00%) |  |
| Widowed | 71 (32.42%) | 62 (32.80%) | 9 (30.00%) |  |
| Other | 10 (4.57%) | 9 (4.76%) | 1 (3.33%) |  |
| family_history, n (p%) |  |  |  | 0.539 |
| Negative | 150 (68.49%) | 128 (67.72%) | 22 (73.33%) |  |
| Positive | 69 (31.51%) | 61 (32.28%) | 8 (26.67%) |  |
| income, n (p%) |  |  |  | 0.766 |
| ＜1000 | 7 (3.20%) | 7 (3.70%) | 0 (0.00%) |  |
| 1000-3000 | 2 (0.91%) | 2 (1.06%) | 0 (0.00%) |  |
| 3000-5000 | 13 (5.94%) | 11 (5.82%) | 2 (6.67%) |  |
| ＞5000 | 22 (10.05%) | 18 (9.52%) | 4 (13.33%) |  |
| NA | 175 (79.91%) | 151 (79.89%) | 24 (80.00%) |  |
| diagnosis, n (p%) |  |  |  | 0.428 |
| Depression | 84 (38.36%) | 71 (37.57%) | 13 (43.33%) |  |
| Mania | 1 (0.46%) | 1 (0.53%) | 0 (0.00%) |  |
| Bipolar Disorder | 103 (47.03%) | 87 (46.03%) | 16 (53.33%) |  |
| Mood Disorder | 31 (14.16%) | 30 (15.88%) | 1 (3.33% |  |
| Onset polarity, n (p%) |  |  |  | 0.320 |
| Depression | 76 (34.70%) | 68 (35.98%) | 8 (26.67%) |  |
| Mania | 143 (65.30%) | 121 (64.02%) | 22 (73.33%) |  |

Supplemental Table 4. Basic information of Train group

| Characteristics | Overall | SB-N | SB | p-value |
| --- | --- | --- | --- | --- |
|  | N = 880 | N = 781 | N = 99 |  |
| age, median (p25 - p75) | 31 (21 - 46) | 32 (21 - 46) | 26 (19 - 39) | 0.001 |
| hospitalization_frequency, median (p25 - p75) | 1(1 - 2) | 1(1 - 2) | 1 (1 - 2) | 0.867 |
| gender, n (p%) |  |  |  | 0.008 |
| Male | 449 (51.02%) | 411 (52.62%) | 38 (38.38%) |  |
| previous_sa, n (p%) |  |  |  | <0.001 |
|  | 405 (46.02%) | 334 (42.77%) | 71 (71.72%) |  |
| previous_sb, n (p%) |  |  |  | <0.001 |
|  | 160 (18.18%) | 125 (16.01%) | 35 (35.35%) |  |
| previous_nssi, n (p%) |  |  |  | 0.007 |
|  | 126 (14.32%) | 103 (13.19%) | 23 (23.23%) |  |
| marriage, n (p%) |  |  |  | 0.517 |
| Single | 237 (26.93%) | 216 (27.66%) | 21 (21.21%) |  |
| Married | 323 (36.70%) | 280 (35.85%) | 43 (43.43%) |  |
| Divorced | 4 (0.45%) | 4 (0.51%) | 0 (0.00%) |  |
| Widowed | 280 (31.82%) | 249 (31.88%) | 31 (31.31%) |  |
| Other | 36 (4.09%) | 32 (4.10%) | 4 (4.04%) |  |
| family_history, n (p%) |  |  |  | 0.770 |
| Negative | 629 (71.48%) | 557 (71.32%) | 72 (72.73%) |  |
| Positive | 251 (28.52%) | 224 (28.68%) | 27 (27.27%) |  |
| income, n (p%) |  |  |  | 0.593 |
| ＜1000 | 38 (4.32%) | 36 (4.61%) | 2 (2.02%) |  |
| 1000-3000 | 4 (0.45%) | 4 (0.51%) | 0 (0.00%) |  |
| 3000-5000 | 38 (4.32%) | 32 (4.10%) | 6 (6.06%) |  |
| ＞5000 | 97 (11.02%) | 87 (11.14%) | 10 (10.10%) |  |
| NA | 703 (79.89%) | 622 (79.64%) | 81 (81.82%) |  |
| diagnosis, n (p%) |  |  |  | 0.001 |
| Depression | 338 (38.41%) | 284 (36.36%) | 54 (54.55%) |  |
| Mania | 8 (0.91%) | 7 (0.90%) | 1 (1.01%) |  |
| Bipolar Disorder | 394 (44.77%) | 354 (45.33%) | 40 (40.40%) |  |
| Mood Disorder | 140 (15.91%) | 136 (17.41%) | 4 (4.04%) |  |
| Onset polarity, n (p%) |  |  |  | <0.001 |
| Depression | 279 (31.70%) | 267 (34.19%) | 12 (12.12%) |  |
| Mania | 601 (68.30%) | 514 (65.81%) | 87 (87.88%) |  |

Supplemental Table 5. Details of hyperparameter for ML models.

| Model | Hyperparameter |
| --- | --- |
| AB | Maximum number of estimators: 100, Learning rate: 0.1, Algorithm: ‘SAMME.R’. |
| CatBoost | Evaluation algorithm: Logloss, Maximum depth of the tree: 10, Iteration number:100. |
| DT | Function to measure the quality of a split: ‘gini’, Maximum depth of the tree: 3. |
| GBDT | Number of boosting stages: 100, Learning rate: 0.1, Maximum depth: 1, criterion: ‘friedman_mse’. |
| LightGBM | Weak learner type: GBDT, Learning rate: 0.1. |
| Logistic regression | Algorithm: ‘LBFGS’, Maximum number of estimators: 100, Stopping criteria:0.001. |
| NB | Criterion: Bernoulli, Additive Laplace/Lidstone: 1.0. |
| RF | Number of trees: 100, Maximum depth of the tree: 3, Minimum number of samples required at a leaf node: 2. |
| SVM | Categories of kernel functions: ‘poly’, Regularization parameter: 1, Degree of the polynomial kernel function: 1. |
| XGBoost | Weak learner type: GBtree, Random number: 1. |

Note: ML, machine learning; AB, AdaBoost; DT, decision tree; GBDT, Gradient Boosting Decision Tree; NB, Naive Bayes; RF, random forest; SVM, support vector machine.

Supplemental Table 6. Variables selected by the Elastic Net regression

| Coef Name | coeff_lamda |
| --- | --- |
| (Intercept) | -2.50414112194806 |
| gender | 0.0638736339416106 |
| age | -0.00792436513977091 |
| previous_sa | 0.422483003493687 |
| previous_sb | 0.457057656371998 |
| previous_nssi | 0.081390326965727 |
| income | 0.000505839130195215 |
| diagnosis | -0.0938627032593118 |
| hospitalization_frequency | -0.00431535875799704 |
| mania_depression | 0.461132083015128 |
| hospitalizationdays | 0.000494483680810291 |
| creatinine | -0.00235933773392002 |
| ACTH | -0.00320151047305485 |
| cortisol | 0.000985910876078054 |
| testosterone | -0.000116078777398293 |

Supplemental Table 7. Variance Inflation Factor (VIF) Analysis for Multicollinearity Assessment of Predictor Variables

| Variable Name | GVIF | Df | GVIF^(1/(2*Df)) |
| --- | --- | --- | --- |
| cerebrovascular_disease | 1.00000017219027 | 1 | 1.00000008609513 |
| family_history | 1.11226204069579 | 1 | 1.05463834592517 |
| TSH | 1.14577803026262 | 1 | 1.0704102158811 |
| D_Dimer | 1.14762115864364 | 1 | 1.07127081480065 |
| psychotic_symptoms | 1.17864209690523 | 1 | 1.08565284364074 |
| FT4 | 1.193718945079 | 1 | 1.09257445745313 |
| leu | 1.21346368354522 | 1 | 1.101573276521 |
| digestive_system_diseases | 1.21555055431163 | 1 | 1.10252009247525 |
| urinary_ketone_body | 1.23193633824813 | 1 | 1.10992627604185 |
| hospitalizationdays | 1.24218996829649 | 1 | 1.11453576357894 |
| progesterone | 1.24275196653343 | 1 | 1.11478785718783 |
| blooddisease | 1.24297095178393 | 1 | 1.11488607121263 |
| VB12 | 1.25544826886338 | 1 | 1.12046787944295 |
| prolactin | 1.26539216688569 | 1 | 1.12489651385613 |
| respiratory_disease | 1.28490560474385 | 1 | 1.13353676814819 |
| homocysteine | 1.32097672663105 | 1 | 1.14933751641154 |
| hospitalization_frequency | 1.36327059402537 | 1 | 1.16759179254796 |
| city | 1.39576004829073 | 1 | 1.18142289138595 |
| urban_rural | 1.40369786949178 | 1 | 1.18477756118682 |
| folic_acid | 1.41093595869204 | 1 | 1.18782825302821 |
| WBC | 1.41155563468406 | 1 | 1.18808906849784 |
| T4 | 1.44432041938283 | 1 | 1.20179882650252 |
| previous_nssi | 1.45515013310086 | 1 | 1.20629603874872 |
| newly_diagnosed_hypothyroidism | 1.46716876969922 | 1 | 1.21126742286715 |
| c_reactive_protein | 1.47004788464158 | 1 | 1.21245531243076 |
| estradiol | 1.47219136015092 | 1 | 1.21333893045221 |
| ACTH | 1.48354411032065 | 1 | 1.21800825544027 |
| income | 1.52994853057048 | 4 | 1.05459231591904 |
| previous_sa | 1.54714181106187 | 1 | 1.24384155384111 |
| mania_depression | 1.55583136292313 | 1 | 1.24732969295336 |
| previous_sb | 1.55807432136391 | 1 | 1.24822847322271 |
| hypertension | 1.57886122711721 | 1 | 1.25652744781688 |
| T3 | 1.62771629218734 | 1 | 1.27581985099282 |
| FT3 | 1.63658190986498 | 1 | 1.27928961141134 |
| C4 | 1.68355607023526 | 1 | 1.2975191984072 |
| history_of_thyroid_diseases | 1.68829582604547 | 2 | 1.13988788183684 |
| total_protein | 1.68831818300277 | 1 | 1.29935298629848 |
| hyperlipemia | 1.69018688843456 | 1 | 1.30007187818003 |
| ESR | 1.69459618825271 | 1 | 1.30176656442417 |
| cortisol | 1.70728417332344 | 1 | 1.30663084814474 |
| Ca | 1.7860412250174 | 1 | 1.33642853344928 |
| CKMB | 1.86225737021319 | 1 | 1.3646455108244 |
| abnormal_liver_function | 1.93726149244728 | 1 | 1.39185541362861 |
| uric_acid | 1.94458935171807 | 1 | 1.39448533578452 |
| triglyceride | 1.96712228734933 | 1 | 1.4025413674289 |
| low_density_lipoprotein | 10.9929355445269 | 1 | 3.31555961257325 |
| cholesterin | 12.8125393557563 | 1 | 3.57946076326537 |
| diabetes | 2.00351288715122 | 1 | 1.41545501064189 |
| BMI | 2.17171336383788 | 1 | 1.47367342509726 |
| glycosylated_hemoglobin | 2.17949497303128 | 1 | 1.47631127240541 |
| high_density_lipoprotein | 2.27564784088617 | 1 | 1.50852505477575 |
| creatinine | 2.32341512224863 | 1 | 1.52427527771352 |
| cardiovasculardisease | 2.41811167120704 | 2 | 1.24700756545644 |
| diagnosis | 2.43830896916529 | 4 | 1.11785659156463 |
| CK | 2.68009701385428 | 1 | 1.63710018442803 |
| C3 | 2.79139345367192 | 1 | 1.67074637622588 |
| occupation | 2.93322796685203 | 8 | 1.06956975500682 |
| somaticdisease | 3.10428977321262 | 1 | 1.76189947874804 |
| marriage | 3.24099228537089 | 4 | 1.15833652180134 |
| ALT | 3.31979352190762 | 1 | 1.82203005516035 |
| testosterone | 3.70291543772665 | 1 | 1.92429608889242 |
| age | 3.80969352266924 | 1 | 1.951843621469 |
| AST | 4.02623956679531 | 1 | 2.00654916879585 |
| gender | 5.78726512699068 | 1 | 2.40567352876293 |
